# A Study on the Clinical Profile of Patients Presenting With Dengue Fever and the Precision Prediction of Dengue Severity Using Platelet Count at Presentation

**DOI:** 10.7759/cureus.63255

**Published:** 2024-06-26

**Authors:** Vikram B Vikhe, Devansh Khandol, Ahsan A Faruqi, Avani Reddy

**Affiliations:** 1 General Medicine, Dr. D. Y. Patil Medical College, Hospital and Research Centre, Dr. D. Y. Patil Vidyapeeth, Pune, IND

**Keywords:** dengue shock syndrome (dss), severe dengue fever, platelet count (plt), thrombocytopenia, dengue fever (df)

## Abstract

Background

The most prevalent arbovirus infection in the world, dengue, has become a serious public health issue. This study aims to examine the clinical characteristics of individuals who present with dengue fever and use platelet count prediction to estimate the severity of dengue.

Materials and methods

This observational, cross-sectional study was conducted at Dr. D. Y. Patil Medical Hospital, Pune, Maharashtra, India, from February 2022 to May 2024. A total of 100 patients older than 12 years old who had dengue fever (presenting within three days of the first symptom) and were dengue NS1 antigen-positive in the laboratory were included. Patients under 12 years of age and pregnant women were excluded. Also excluded were patients with a history of prior dengue infection and patients on medications causing thrombocytopenia, such as antiplatelets (aspirin). Written informed consent was obtained from each patient. For adolescent boys and girls aged 13-18, consent was obtained from a parent or legal guardian along with the adolescent’s assent. Data were collected through physical examinations and laboratory investigations. Statistical analysis was performed using IBM SPSS Statistics for Windows, Version 20 (Released 2011; IBM Corp., Armonk, New York), with descriptive statistics and tests for nonparametric data, setting the significance at p<0.05.

Results

The average age of the study participants was 29.48 ± 10.62 years, with 24% in the 0-20 year age group, 36% in the 21-30 age group, 24% in the 31-40 age group, 12% in the 41-50 age group, 3% in the 51-60 age group, and 1% in the 61-70 age group. Men comprised 65% of the population, with 35% being women. Weakness was the most prevalent symptom, followed by nausea and fever with chills. Patients with dengue fever without warning indications had a median platelet count of 114,000/µL upon admission; those with dengue fever with warning signs had a median count of 35,500/µL; and those with severe dengue had a median count of 25,000/µL. These distinctions attained statistical significance, underscored by p-values of <0.001. The predictive model for severe dengue using platelet count on presentation demonstrated a robust capacity to anticipate severe dengue with a noteworthy association (p<0.04), indicating an increased risk of severe dengue with a lower platelet count (<25,000/µL, odds ratio (OR) 7.5).

Conclusion

Dengue was more common in the young population, with a predominance of male patients. Weakness was the most common symptom. Patients with a platelet count less than 25,000/µL had 7.5 times more odds of developing severe dengue.

## Introduction

In tropical and subtropical areas worldwide, dengue fever, a mosquito-borne virus, remains a serious public health concern [1]. Endemic in over 100 countries, primarily in Southeast Asia, the Western Pacific, the Americas, and Africa, dengue is spread mainly by *Aedes aegypti* and *Aedes albopictus* mosquitoes. Factors such as urbanization, increased travel, and climate change contribute to its rising incidence and expanding geographic distribution.

The clinical manifestations of dengue fever vary widely, ranging from mild to severe forms. Mild dengue fever typically presents with flu-like symptoms such as high fever, severe headache, retro-orbital pain, muscle and joint pain, rash, and mild bleeding manifestations such as nosebleeds and gum bleeding. Severe forms of the disease, such as dengue hemorrhagic fever (DHF) and dengue shock syndrome (DSS), can lead to significant morbidity and mortality. These severe forms are characterized by plasma leakage, leading to fluid accumulation in body cavities, severe bleeding, and organ impairment. Vasoactive inflammatory mediators that macrophages excrete produce vascular leakage, which can lead to shock in cases of severe vascular leakage. Perivascular edema and swelling of endothelial cells are possible. Without timely and appropriate medical intervention, severe dengue can progress to shock, organ failure, and death. Early recognition and prompt management are crucial to reducing the fatality rates associated with severe dengue. Identifying early predictors of disease severity is essential for effective clinical management.

A complex relationship between body mass index (BMI) and the severity of dengue fever suggests that BMI is a significant factor in determining the clinical outcomes of the disease. Research indicates that individuals with a normal BMI are more susceptible to severe dengue complications compared to those at the extremes of the BMI spectrum. It has been found that people with normal BMI are more likely to develop severe DHF or DSS, possibly due to better immune responses that inadvertently increase the severity of the symptoms.

Among clinical and laboratory parameters, platelet count is a crucial marker for managing and prognosing dengue patients. Thrombocytopenia, or low platelet counts, is common in dengue and associated with a higher risk of bleeding and severe disease [2]. The dengue virus directly affects the bone marrow, leading to decreased platelet production and increasing the destruction of platelets in the bloodstream. Monitoring platelet count helps identify high-risk patients and guide clinical decisions, although its predictive value at presentation requires further study [3].

By analyzing patient data, the study seeks to optimize early diagnostic strategies and develop tailored intervention protocols, enhancing clinical outcomes [4]. Correlating initial platelet counts with clinical outcomes will help healthcare providers stratify patients by risk, improving resource allocation and patient care.

Dengue is classified into severe dengue and dengue with or without warning signs [5]. The World Health Organization (WHO) uses this classification to facilitate clinical management. Warning signs include abdominal pain, persistent vomiting, fluid accumulation, mucosal bleeding, lethargy, restlessness, liver enlargement, and hematocrit increase with a rapid platelet count decrease. Recognizing these signs is crucial for early intervention to prevent severe dengue, guiding hospital admission and intensive care prioritization to improve outcomes and reduce mortality.

Aims and objectives

This research aims to examine the clinical characteristics of dengue patients and assess the accuracy of platelet count at presentation in predicting disease severity.

## Materials and methods

Study design and setting

This observational, cross-sectional study was conducted at Dr. D. Y. Patil Medical College, Hospital and Research Centre (DYPMCH), Pune, Maharashtra, India. The study included 100 patients with a primary diagnosis of dengue fever who were hospitalized between February 2022 and May 2024, with each participant undergoing a thorough clinical assessment and investigation. The study received approval from the Institutional Ethics Committee of DYPMCH (approval number IESC/PGS/2022/11, dated January 28, 2022).

Inclusion criteria

The study included patients older than 12 years who had dengue fever (presenting within three days of the first symptom) and were dengue NS1 antigen-positive in the laboratory.

Exclusion criteria

Patients under the age of 12 and pregnant women were excluded from the study. Patients with a history of prior dengue infection and patients on medications causing thrombocytopenia, such as antiplatelets (aspirin), were also excluded.

Sample size

Based on the study conducted by Avila-Aguero et al., estimating the proportion of retro-orbital pain at 33%, an acceptable difference of 8%, and a 95% confidence interval, the minimum sample size was calculated to be 100 using WinPepi software, version 11.38 (J. H. Abramson, Brixton Health, United Kingdom) [6].

Data and sample collection

A detailed clinical history was taken from all patients using a pretested pro forma, focusing on symptoms of dengue fever. Physical examinations (including calculating the BMI by the metric system (kg/m^2^) and classifying as per Asian Criteria BMI-cutoff) and necessary investigations were performed, such as dengue profile; hemogram, which was conducted using the DxH 900 analyzer (Beckman Coulter, Brea, California), based on the Coulter Principle. The Coulter Principle is used to directly count and size cell volume by detecting and measuring changes in electrical resistance when a particle (such as a cell) in a conductive liquid passes through a small aperture; renal (RFT) and liver function tests (LFT); and serum electrolytes (analyzed using the ARCHITECT c8000 (Abott, Green Oaks, Illinois) using spectrophotometric measurement methods. The serum used in this study was centrifuged at 4,000 rpm for 20 minutes. The serum was excluded in case of being lysed, lipemic, or icteric). The sample collection procedure began by identifying and explaining it to the patient. Supplies were gathered, hand hygiene was performed, and gloves were put on. The patient was positioned, a tourniquet was applied, and a venipuncture site was selected. The site was cleaned with an antiseptic and allowed to dry. The needle and holder were assembled, and the needle was inserted into the vein. Blood was collected in an EDTA (lavender) vacutainer for CBC and in a plain (red) vacutainer for dengue profile, LFT, RFT, and serum electrolytes, following the correct order of draw to avoid contamination. The tourniquet was released, the needle was withdrawn, and pressure was applied to the site. The needle was disposed of in a sharps container, the tubes were labeled, and the puncture site was bandaged. The patient was checked for well-being, samples were transported and stored as required, and the procedure was documented in the patient’s record.

The diagnosis was based on demonstrating that reciprocal IgG or IgM antibody titers to one or more dengue viral proteins had changed by a factor of four or more in matched serum samples using the J Mitra dengue ELISA kit (New Delhi, India), which is designed for in vitro qualitative detection of dengue NS1 antigen in human serum or plasma and is used as a screening test for testing of collected blood samples suspected for dengue. The kit detects all four subtypes: DEN1, DEN2, DEN3, and DEN4 of the dengue virus, based on the Direct Sandwich ELISA principle. It exhibits a significant reduction in the window period and a longer shelf life of 24 months at 2-8 °C. The sensitivity is 99.50%, and the specificity is 100%. It has a convenient pack size, containing 96 tests. Its principle involves coating the microwells with anti-dengue NS1 antibodies with high reactivity for dengue NS1 antigen. The samples are added to the wells, followed by the addition of an enzyme conjugate (monoclonal anti-dengue NS1 antibodies linked to Horseradish peroxidase (HPRO)). A sandwich complex is formed in the well wherein dengue NS1 (from serum sample) is "trapped" or "sandwiched" between the antibody and antibody HRPO conjugate. Unbound conjugate is then washed off with a wash buffer. The amount of bound peroxidase is proportional to the concentration of dengue NS1 antigen present in the sample. Upon addition of the substrate buffer and chromogen, a blue color develops. The intensity of the developed blue color is proportional to the concentration of dengue NS1 antigen in the sample. The enzyme-substrate reaction is halted by the addition of a stop solution. A yellow color develops, which is finally read at 450 nm. The biological reference interval for the test result is as follows: Negative: <0.90 ratio; Equivocal: 0.90 to <1.10 ratio; and Positive: ≥1.10 ratio.

Consent

Written informed consent was obtained from each patient. For adolescent boys and girls aged 13-18, consent was obtained from a parent or legal guardian along with the adolescent’s consent. The study recruited 100 patients, explained the procedure and purpose to them, and obtained informed consent from the patient or legal guardian.

Statistical analysis

Data were entered into a Microsoft Excel 2010 (Microsoft Corporation, Redmond, Washington) spreadsheet and imported into IBM SPSS Statistics for Windows, Version 20 (Released 2011; IBM Corp., Armonk, New York) for analysis. Descriptive statistics included standard deviations, averages, and percentages, while the Kruskal-Wallis test, odds ratio (OR), and Chi-square test assessed the statistical significance of nonparametric data distribution, with a p-value and confidence interval set at 5% and 95%, respectively.

## Results

Gender distribution across the severity of dengue

The distribution of gender concerning the severity of dengue was analyzed, with severity categorized into three levels: dengue fever without warning signs, dengue fever with warning signs, and severe dengue (Table 1). Among the female participants, nine patients (27%) had dengue fever without warning signs, 25 (42%) had dengue fever with warning signs, and one (14%) had severe dengue, making a total of 35 female participants. Among the male participants, 24 (73%) had dengue fever without warning signs, 35 (58%) had dengue fever with warning signs, and six (86%) had severe dengue, summing up to 65 male participants. The total number of participants in the study was 100, with 33 having dengue fever without warning signs, 60 having dengue fever with warning signs, and seven having severe dengue.

**Table 1:** Distribution of gender across the severity of dengue among the study participants (N = 100) n: number; %: percentage

Sex	Severity of Dengue			Total
	Dengue fever without warning signs	Dengue fever with warning signs	Severe dengue	
Female (n, %)	9 (27%)	25 (42%)	1 (14%)	35
Male (n, %)	24 (73%)	35 (58%)	6 (86%)	65
Total	33	60	7	100

Age distribution across the severity of dengue

The relationship between age and dengue severity was investigated across different age groups (0-20, 21-30, 31-40, 41-50, 51-60, and 61-70). The participants were categorized into dengue fever without warning signs, dengue fever with warning signs, and severe dengue. The distribution across severity categories varied notably among different age groups. The participants in the 0-20 years age group showed a higher proportion in the severe dengue category (three cases) compared to other age groups. The 21-30 age group had the highest number of participants with dengue fever with warning signs (25 cases). The 31-40 age group had a relatively even distribution across the severity categories. The participants aged 41-50 had no severe dengue cases, while those in the 51-60 and 61-70 age groups had very few cases overall, with none classified as severe dengue. These findings (Table 2) suggest that age may serve as a critical factor in determining the severity of dengue, emphasizing the importance of age-specific considerations in clinical management and public health interventions.

**Table 2:** Distribution of age across the severity of dengue among the study participants (N = 100) n: number; %: percentage

Age	Severity of Dengue			Total
	Dengue fever without warning signs	Dengue fever with warning signs	Severe dengue	
0-20 (n, %)	11 (33%)	10 (17%)	3 (43%)	24
21-30 (n, %)	9 (27%)	25 (42%)	2 (29%)	36
31-40 (n, %)	8 (24%)	14 (23%)	2 (29%)	24
41-50 (n, %)	4 (12%)	8 (13%)	0 (0%)	12
51-60 (n, %)	1 (3%)	2 (3%)	0 (0%)	3
61-70 (n, %)	0 (0%)	1 (2%)	0 (0%)	1
Total	33	60	7	100

BMI distribution across the severity of dengue

The investigation into the relationship between BMI (classified as per Asian cutoff criteria for BMI classification) and the severity of dengue fever revealed significant patterns within different BMI categories. The participants were divided into five BMI ranges: <18.5, 18.5-22.9, 23-24.9, 25-29.9, and ≥30 (Table 3). The findings demonstrated that individuals within the normal BMI range (18.5-22.9) had the highest overall number of dengue cases, with a notable 57% of severe dengue cases occurring in this group. This suggests a significant correlation between a normal BMI and the likelihood of experiencing severe dengue outcomes. Conversely, the participants with a BMI of <18.5 had no cases of severe dengue but showed a higher proportion (22%) of dengue with warning signs. Similarly, the BMI ≥30 group also had no severe dengue cases but had 18% of cases without warning signs and 10% with warning signs, indicating less severity overall. The 23-24.9 BMI range showed a mixed distribution, with 12% of cases without warning signs, 17% with warning signs, and 29% severe dengue, while the 25-29.9 BMI range had 6% without warning signs, 26% with warning signs, and 14% severe dengue. These patterns highlight that while individuals with a normal BMI are more prone to severe dengue, those at the extremes of BMI, both underweight and obese, tend to exhibit different distributions of dengue severity. This emphasizes the necessity for tailored clinical assessments and public health strategies that consider BMI for effective dengue management.

**Table 3:** Distribution of body mass index (BMI) across the severity of dengue among the study participants (N = 100) Grading of BMI (Asian criteria): below 18.5: underweight; 18.5–22.9: normal; 23.0–24.9: overweight; 25.0–29.9: pre-obese; and ≥30: obese n: number; %: percentage; BMI: body mass index

BMI	Severity of Dengue			Total
	Dengue fever without warning signs	Dengue fever with warning signs	Severe dengue	
<18.5 (n, %)	4 (12%)	13 (22%)	0 (0%)	17
18.5-22.9 (n, %)	17 (52%)	15 (25%)	4 (57%)	36
23-24.9 (n, %)	4 (12%)	10 (17%)	2 (29%)	16
25-29.9 (n, %)	2 (6%)	16 (26%)	1 (14%)	19
≥30 (n, %)	6 (18%)	6 (10%)	0 (0%)	12
Total	33	60	7	100

Distribution of the severity of dengue among the study participants

**Figure 1:**
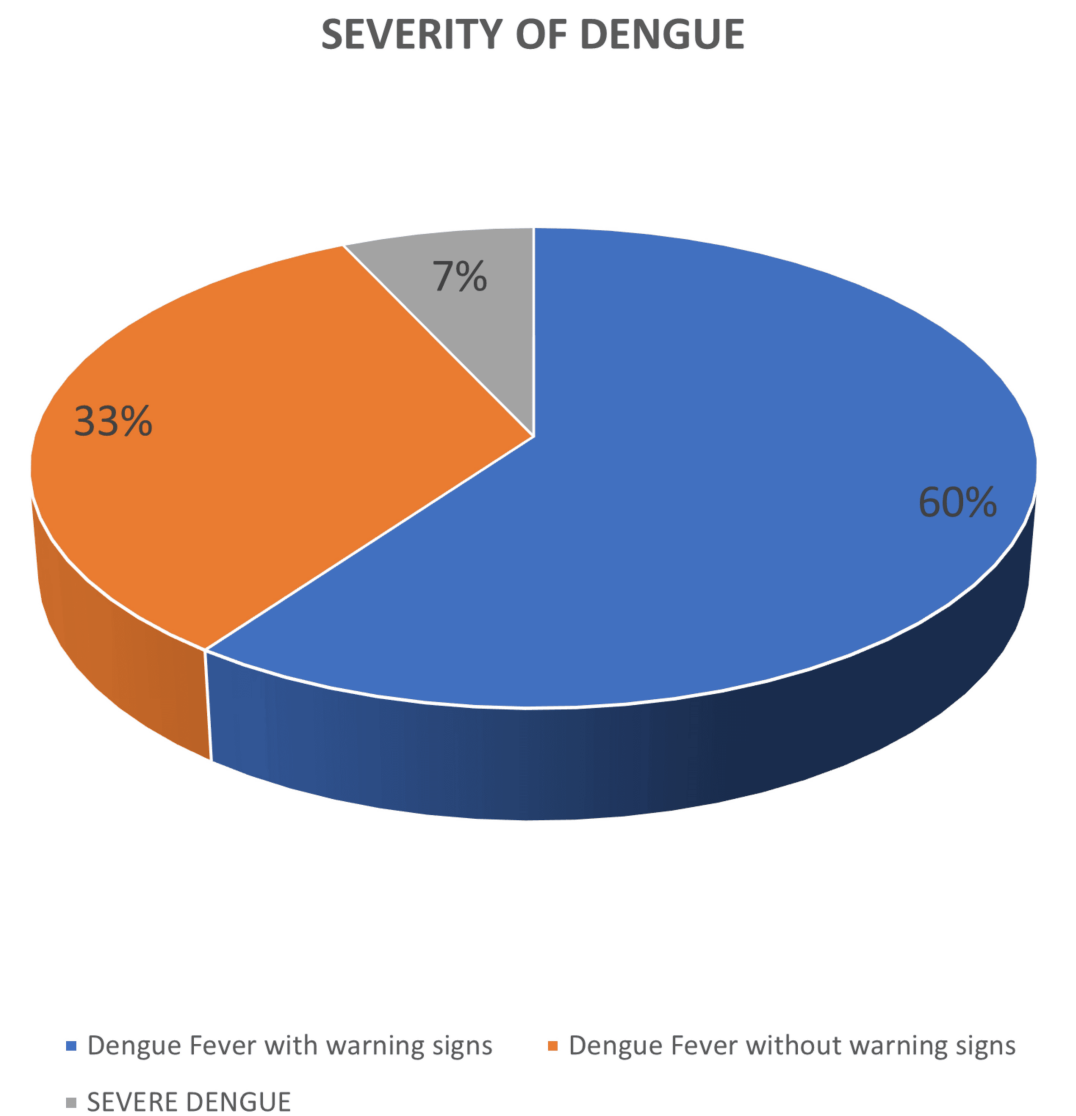
Pie chart showing the distribution of the severity of dengue among the study participants (N=100) Based on the data, 33% (33 patients) had dengue fever without warning signs, 60% (60 patients) had dengue fever with warning signs, and 7% (seven patients) had severe dengue.

The severity of dengue cases among the participants varied significantly (Figure 1). The cases were categorized into dengue fever without warning signs (33%), dengue fever with warning signs (60%), and severe dengue (7%). This distribution indicates that while most participants experienced dengue fever with or without warning signs, a smaller proportion suffered from severe forms of the illness.

Summary of routine laboratory investigations

The mean values ± standard deviations of various routine laboratory investigations conducted among the study participants (N=100) were summarized (Table 4). Key parameters include hemoglobin (11.73 ± 1.66 g/dL), total leukocyte count (5016.4 ± 3009.81 cells/μL), neutrophil-to-lymphocyte ratio (3.1 ± 3.26), and bilirubin levels total (1.47 ± 1.73 mg/dL), direct (0.88 ± 1.04 mg/dL), and indirect (0.59 ± 0.69 mg/dL). Enzyme levels measured were serum glutamic pyruvic transaminase (131.95 ± 192.57 U/L) and serum glutamic oxaloacetic transaminase (109.21 ± 154.05 U/L), along with alkaline phosphatase (169.37 ± 46.52 U/L). Metabolic indicators included urea (30.11 ± 11.46 g/dL), serum creatinine (1.02 ± 0.27 g/dL), and electrolyte levels-serum sodium (138.2 ± 3.87 mmol/L), potassium (4.34 ± 0.65 mmol/L), and chloride (99.75 ± 2.65 mmol/L). These results provide a detailed overview of the participant's health status, which is crucial for clinical assessment and potential interventions.

**Table 4:** Summary of routine laboratory investigations among the study participants (N=100) g/dL: gram per deciliter; /µL: per microliter; mg/dL: milligram per deciliter; U/L: unit per liter; mmol/dL: millimole per liter; std. deviation: standard deviation; TLC: total leukocyte count; NLR: neutrophil-to-lymphocyte ratio; TB: total bilirubin; DB: direct bilirubin; IB: indirect bilirubin; SGOT: serum glutamic oxaloacetic transaminase; SGPT: serum glutamic pyruvic transaminase; ALP: alkaline phosphatase

Variable	Mean ± Std. Deviation	Variable	Mean ± Std. Deviation
Hemoglobin (g/dL)	11.73 ± 1.66	SGPT (U/L)	131.95 ± 192.57
TLC (/μL)	5016.4 ± 3009.81	ALP (U/L)	169.37 ± 46.52
NLR	3.1 ± 3.26	Urea (g/dL)	30.11 ± 11.46
TB (mg/dL)	1.47 ± 1.73	Creatinine (g/dL)	1.02 ± 0.27
DB (mg/dL)	0.88 ± 1.04	Serum sodium (mmol/L)	138.2 ± 3.87
IB (mg/dL)	0.59 ± 0.69	Serum potassium (mmol/L)	4.34 ± 0.65
SGOT (U/L)	109.21 ± 154.05	Serum chloride (mmol/L)	99.75 ± 2.65

Distribution of clinical features at presentation across different severities

Clinical features observed in patients with dengue fever were categorized by severity (N=100), along with associated p-values assessing the significance of these associations (Table 5). Fever with chills was reported in 39% of cases, with no statistically significant difference across severity groups (p = 0.124). Retro-orbital pain occurred in 30% of patients, with no significant variation by severity (p = 0.655). Weakness was prevalent in 75% of cases, significantly more common in milder forms (dengue fever without warning signs) compared to severe dengue (p = 0.03). Nausea and vomiting were observed in 41% and 26% of cases, respectively, with p-values indicating no significant differences across severity groups (p = 0.06 for nausea, p = 0.44 for vomiting). Rash and conjunctivitis were less frequent (<10%) and did not vary significantly across severity groups (p = 0.781 for rash, p = 0.329 for conjunctivitis). Severe symptoms, such as requiring oxygen on admission, were noted in 16% of cases, significantly more common in severe dengue compared to other severity categories (p = 0.006). These p-values help interpret the association between clinical features and the severity of dengue, highlighting which symptoms may be more indicative of severe illness.

**Table 5:** Distribution of clinical features at presentation seen in patients with dengue fever across different severities

Clinical Features	Total (N=100)	Severity of Dengue			p-value
		Dengue fever without warning signs (N=33)	Dengue fever with warning signs (N=60)	Severe dengue (N=7)	
Fever with chills	39 (39%)	10 (30%)	24 (40%)	5 (71%)	0.124
Retro-orbital pain	30 (30%)	8 (24%)	20 (33%)	2 (29%)	0.655
Weakness	75 (75%)	30 (90%)	40 (67%)	5 (71%)	0.03
Nausea	41 (41%)	10 (30%)	30(50%)	1 (14%)	0.06
Vomiting	26 (26%)	11 (33%)	14 (23%)	1 (14%)	0.44
Rash	6 (6%)	2 (6%)	4(7%)	0 (0%)	0.781
Conjunctivitis	7 (7%)	4 (12%)	3 (5%)	0 (0%)	0.329
Severe pain in the back	28 (28%)	12 (36%)	14 (23%)	2 (29%)	0.408
Severe pain in the muscles	36 (36%)	12 (36%)	23 (38%)	1 (14%)	0.455
Required oxygen on admission	16 (16%)	0 (0%)	14 (23%)	2 (29%)	0.006

Association between platelet count and the severity of dengue

The Kruskal-Wallis test was employed to compare platelet counts on admission (per microliter) across different severities of dengue among the study participants (with the first symptom presenting on the first day in 26 patients, 32 on the second day, and 42 on the third day, respectively). The median platelet count for participants with dengue fever without warning signs was 114,000 (interquartile range (IQR): 44,500-164,000). Those with dengue fever with warning signs had a median platelet count of 35,500 (IQR: 17,000-75,500), while participants with severe dengue presented a median platelet count of 25,000 (IQR: 17,000-45,000). The p-value for this comparison was <0.001, indicating a statistically significant difference in platelet counts across the severity levels of dengue. This statistical analysis underscores the substantial variation in platelet counts based on the severity of the disease, highlighting its potential diagnostic and prognostic value in clinical management (Table 6).

**Table 6:** Kruskal-Wallis test conducted on the platelet count at admission (nonparametric data) across the severity of dengue

Kruskal-Wallis Test		Platelet Count on Admission (per Microliter)	
		Median	Interquartile range
Severity of dengue	Dengue fever without warning signs	114,000.00	44,500-164,000
	Dengue fever with warning signs	35,500	17,000-75,500
	Severe dengue	25,000	17,000-45,000
	p-value	<0.001	

**Figure 2:**
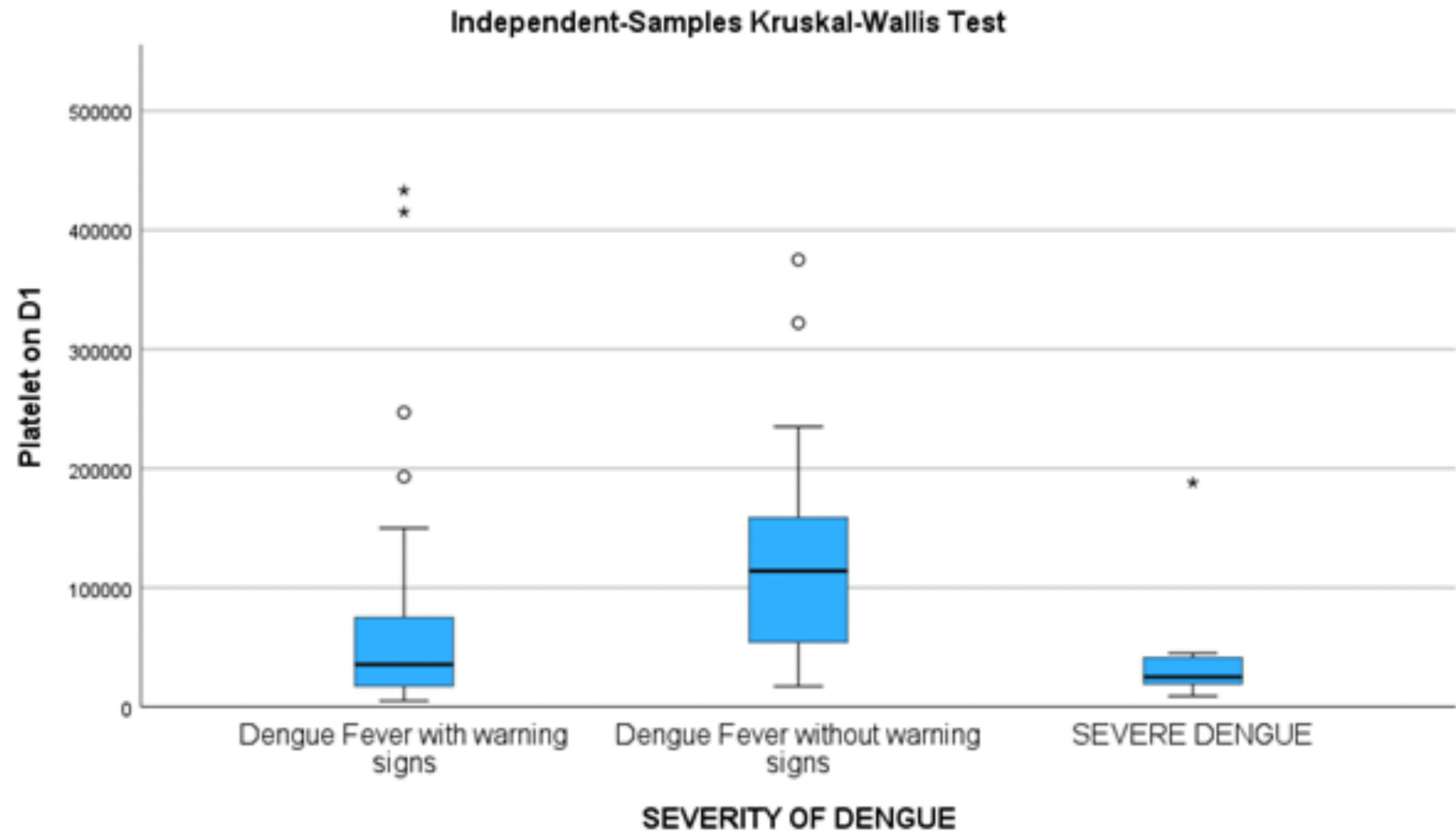
Whisker plot showing the median values of platelet count with interquartile range, using independent-samples Kruskal-Wallis test Platelet on D1: platelet count on day one.

The Whisker plot (Figure 2) displays the IQR with the median values of platelet count for all three groups of patients according to severity.

Prediction of the severity of dengue using platelet count at presentation

Using a cutoff of platelet count less than 25,000/μL (as per the median value of platelet count in patients with severe dengue), a significant association was found, indicating an increased risk of severe dengue with diminished platelet counts (<25,000/μL, OR 7.5). This finding underscores the critical role of platelet counts as a prognostic marker for dengue severity, with patients below this threshold having 7.5 times higher odds of progressing to severe dengue (p < 0.04). This insight highlights the importance of platelet count as an early indicator in clinical assessment, emphasizing its utility in identifying individuals at higher risk of severe dengue and guiding early intervention strategies to mitigate adverse outcomes.

## Discussion

This study offers a comprehensive investigation into the clinical characteristics of patients presenting with dengue fever, coupled with a meticulous evaluation of platelet count's predictive utility in determining disease severity. The demographic profile of the study participants portrays a mean age of 29.48 ± 10.62 years, with a notable concentration in the 21-30 age bracket. This age distribution corroborates existing epidemiological trends, often highlighting a higher incidence of dengue fever among young adults. The gender distribution in this study demonstrates a pronounced male predominance, with 65% of cases being male and 35% female. Such gender disparities in dengue incidence have been observed in various epidemiological studies and may reflect differences in exposure patterns, immune responses, or healthcare-seeking behaviors between genders [7].

Among the clinical manifestations observed in the study cohort, weakness emerged as the most prevalent symptom, followed by nausea and fever with chills. These symptoms are consistent with data published in the literature and match the traditional presentation of dengue fever [4]. The prominence of these symptoms underscores their importance as early indicators of dengue infection and highlights the need for clinicians to maintain a high index of suspicion, particularly in endemic regions.

This study's primary objective was to determine whether platelet count could predict the severity of dengue. The data showed that patients with different severity levels of the condition had significantly different median platelet counts. In particular, patients without warning signs of dengue fever had a median platelet count of 114,000/μL, while patients with severe dengue or warning signs had significantly lower median platelet counts of 25,000/μL and 35,500/μL, respectively. These findings emphasize the strong inverse correlation between platelet count and disease severity, corroborating previous research indicating thrombocytopenia as a hallmark feature of severe dengue [3]. Developing a predictive model for severe dengue using platelet count at presentation is of particular significance. This model demonstrated a robust capacity to anticipate severe dengue, with a noteworthy association between lower platelet counts (<25,000/μL) and an increased risk of severe dengue, as evidenced by an OR of 7.5. These prediction models have enormous potential to direct clinical judgment and resource allocation, allowing medical professionals to recognize high-risk patients early in the course of the disease and implement suitable management measures [2,8].

From a clinical perspective, the results of this study highlight the crucial role of platelet count measurement for early dengue patient triage and care. By leveraging platelet count as a predictive marker, clinicians can stratify patients based on their risk of disease progression, enabling targeted interventions and close monitoring of high-risk individuals. Moreover, the robust statistical significance and high predictive accuracy of the developed model lend credibility to its potential adoption in routine clinical practice, thereby enhancing the precision and efficacy of dengue management protocols.

Moving forward, it is imperative to consider the broader implications of these findings within the context of dengue management and public health interventions. Firstly, the robust predictive capacity of platelet count in identifying patients at risk of severe dengue underscores the importance of routine hematological assessments in suspected cases. Integrating platelet count measurements into standard diagnostic protocols can facilitate early risk stratification and guide clinicians in allocating resources and implementing appropriate treatment strategies. Furthermore, the development and validation of predictive models, such as the one presented in this study, represent significant strides toward enhancing the precision of dengue severity predictions. By leveraging advanced statistical techniques and machine learning algorithms, future iterations of predictive models can incorporate a wider array of clinical and laboratory parameters, further refining their accuracy and utility in clinical practice.

The findings of this research have broader implications for public health campaigns aimed at reducing the incidence of dengue fever beyond just managing individual patients. Using platelet count thresholds to identify high-risk populations can help with targeted interventions such as vector control and community education programs. By prioritizing resources and interventions in areas with a higher prevalence of severe dengue, public health authorities can optimize their efforts to curb disease transmission and reduce the overall impact of dengue outbreaks.

However, several inherent limitations must be acknowledged, which may affect the generalizability and interpretation of the study findings. Firstly, the study's single-center design, conducted at a tertiary care hospital in Western India, limits the diversity of patient demographics and regional variations in dengue presentation, potentially restricting the applicability of findings to broader populations. Secondly, the observational nature of the study introduces inherent biases, such as selection bias and confounding variables, which could influence the accuracy of associations observed, particularly concerning predictive models and ORs. Additionally, the relatively small sample size of 100 laboratory-confirmed cases may limit the statistical power and robustness of the conclusions drawn, particularly in rare outcomes or subgroups. Lastly, the study period from February 2022 to May 2024 may not fully capture seasonal variations in dengue incidence and severity, impacting the temporal relevance of the findings. These limitations underscore the need for caution when interpreting and applying the study's results to clinical practice and public health interventions beyond the study setting. To overcome these constraints, future research should focus on prospective, multicenter studies with larger and more diverse patient populations. Additionally, incorporating other clinical and laboratory parameters, alongside platelet count, into predictive models may further enhance their accuracy and reliability, thereby advancing our understanding of dengue pathophysiology and improving patient outcomes.

While platelet count serves as a valuable prognostic marker, its predictive utility may be influenced by various factors, including patient demographics, comorbidities, and circulating viral strains. Future studies should aim to elucidate the multifaceted interplay between these factors and refine existing predictive models to account for their complexities.

## Conclusions

This study provides compelling evidence supporting the pivotal role of platelet count as a prognostic marker for dengue severity. It not only adds to the existing body of knowledge but also offers valuable insights that guide and improve clinical care techniques for dengue patients by clarifying the complex relationship between platelet dynamics and illness progression. Fever was more common in the younger population, with a male predominance. As the severity of dengue progressed, there was a higher likelihood of requiring oxygen. This study significantly contributes to the field of dengue research by elucidating the prognostic value of platelet count in predicting disease severity. By leveraging platelet count measurements and developing predictive models, clinicians and public health practitioners can enhance their capacity to identify and manage patients at risk of severe dengue, ultimately contributing to improved clinical outcomes and the effective control of dengue outbreaks. Patients with a platelet count less than 25,000/μL had 7.5 times higher odds of progressing to severe dengue.
